# Effects of Cationic and Anionic Surfaces on the Perpendicular and Lateral Forces and Binding of *Aspergillus niger* Conidia

**DOI:** 10.3390/nano13222932

**Published:** 2023-11-11

**Authors:** Kathryn A. Whitehead, Stephen Lynch, Mohsin Amin, Ted Deisenroth, Christopher M. Liauw, Joanna Verran

**Affiliations:** 1 Microbiology at Interfaces, Manchester Metropolitan University, Chester St., Manchester M1 5GD, UK; m.amin@mmu.ac.uk (M.A.); chris.liauw.curvi.hifi@googlemail.com (C.M.L.);; 2 Department of Computing and Mathematics, Manchester Metropolitan University, Chester St., Manchester M1 5GD, UK; s.lynch@mmu.ac.uk; 3 BASF Corporation (Formerly Ciba Speciality Chemicals Inc.), Tarrytown, NY 10591, USA; ted.deisenroth@basf.com

**Keywords:** cationic, anionic, fungal conidia, *Aspergillus*, multifractal analysis, atomic force microscopy force measurements

## Abstract

The binding of conidia to surfaces is a prerequisite for biofouling by fungal species. In this study, *Aspergillus niger* subtypes 1957 and 1988 were used which produced differently shaped conidia (round or spikey respectively). Test surfaces were characterised for their surface topography, wettability, and hardness. Conidial assays included perpendicular and lateral force measurements, as well as attachment, adhesion and retention assays. Anionic surfaces were less rough (*R_a_* 2.4 nm), less wettable (54°) and harder (0.72 GPa) than cationic surfaces (*R_a_* 5.4 nm, 36° and 0.5 GPa, respectively). Perpendicular and lateral force assays demonstrated that both types of conidia adhered with more force to the anionic surfaces and were influenced by surface wettability. Following the binding assays, fewer *A. niger* 1957 and *A. niger* 1988 conidia bound to the anionic surface. However, surface wettability affected the density and dispersion of the conidia on the coatings, whilst clustering was affected by their spore shapes. This work demonstrated that anionic surfaces were more repulsive to *A. niger* 1998 spores than cationic surfaces were, but once attached, the conidia bound more firmly to the anionic surfaces. This work informs on the importance of understanding how conidia become tightly bound to surfaces, which can be used to prevent biofouling.

## 1. Introduction

An ever-increasing global public health concern is that of fungal infections [1]. Resistant microorganisms prevent the efficacy of a number of available medicines, resulting in the persistence and spread of antimicrobial-resistant organisms [2]. *Aspergillus niger* is an opportunistic pathogen that is usually considered to be of low virulence [3]. However, although *Aspergillus fumigatus* is a predominant etiological agent, when clinical samples have been analysed, *Aspergillus flavus* and *A. niger* isolates have both been recovered [4]. In a small number of instances, *A. niger* can colonise the body opportunistically if patients are severely ill or immunosuppressed [5]. People most at risk are those with underlying health problems or a weakened immune system, such as those with chronic lung disease, prior tuberculosis (TB), human immunodeficiency virus, cancer, or diabetes mellitus [1]. When present as a co-species with *Candida albicans*, *A. niger* has been known to cause chronic necrotising semi-invasive pneumonia [6]. *A. niger* can also cause pulmonary infections [7,8], and although rare, necrotizing *A. niger* fungal pneumonia has been reported to be the cause of invasive pulmonary aspergillosis [9], which has also been found following bilateral lung transplantation [3]. In addition, *A. niger* infections have been found in patients with haematological diseases and this fungus constitutes the most frequent agent of otomycosis [10,11,12]. Such infections are of extreme importance, since the treatment of invasive fungal infections involves the use of systemic antifungal drugs which must be used for long periods of time and these may have severe side effects [13].

Fungal transmission can occur because conidia can survive in a number of extreme environments [14]. Additionally, it has been shown that *A. niger* has the ability to form biofilms on polymeric structures [15,16,17], a process which is preceded by spore adhesion. In an attempt to reduce infections arising from contamination in hospitals, a number of potential solutions have been suggested. One such solution is to produce antiadhesive surfaces that are repellent to fungal conidia. Research on antimicrobial surfaces is increasing, but only a few studies have been carried out on antiadhesive surfaces [13]. One advantage of using antiadhesive surfaces is that fungal spores are particularly resistant to many types of antimicrobial and biocidal agents that may be released from antimicrobial surfaces, so this should always be considered in such investigations. 

When fungal binding was compared between different types of surfaces, it was found that fewer studies investigated the effects of anionic polymers on biofouling, compared to cationic surfaces [18,19]. In addition, although there has been some exploration in this context, most studies on cationic surfaces have been carried out using yeast, rather than filamentous fungi or spores [13]. Adherence between spores and/or hyphae and the substratum is a complex process, which is dependent on a number of physicochemical (e.g., charge and hydrophobicity) and surface interactions that are not well understood [20]. In addition to the surface properties, the properties of the conidia, such as the presence of glycoproteins, hydrophobins, carbohydrates, and lipids, also influence their binding behaviour [21].

The methods generally used to determine the antiadhesive efficacy of fungal spore binding to surfaces include counting methods. However, atomic force microscopy (AFM) has allowed for force measurements of individual conidia attachment to a surface [14,22]. This involves using a single sphere or fungal spore, which has been bound to a cantilever, to create a particle probe. The force of the attachment between the conidia and the surface can then be determined. The AFM can also be used to carry out lateral force measurements, which can be used to determine the force needed to remove microorganisms from a surface under liquid [23,24,25]. 

The use of microscopy enables the use of percentage coverage to determine the number of microorganisms bound to a surface [26]. However, the percentage of coverage only yields the total amount of cells across the surface and does not describe the distribution of the microorganisms. MATLAB^®^ is a mathematical package that enables multifractal analysis to be used to describe the dispersion, distribution, or clustering of microorganisms across surfaces [27], since it uses a box-counting method used to calculate fractal dimensions [28,29]. This is important, since determining the distribution and pattern of conidia across surfaces may inform how surface properties affect binding. There has been continuing interest in multifractal analysis for such concepts, and these have been gradually more widely used in a variety of scientific disciplines [29,30,31].

The aim of this work was to determine how the properties of anionic and cationic surfaces influenced the perpendicular and lateral forces on fungal conidia removal, as well as the amount and distribution of binding, in order to determine the antiadhesive nature of the test surfaces.

## 2. Methods and Materials

### 2.1. Spin Coated Surfaces

Silicon wafers (Montco Technologies, Spring City, PA, USA) were used as substrates on which to produce the spin-coated surfaces, using polymers dissolved in 20% tetrahydrofuran (THF). Methacylic acid, polymerised with γ-MPS (3-methacryloxypropyltrimethoxysilane), was used to produce the anionic surface, and a quaternary ammonium functional methacrylate, copolymerised with γ-MPS, was used to produce the cationic surface. The polymerising mixture was dropped onto silicon wafer disks covering them and the samples were spun at 2000 rpm for 10–15 s.

### 2.2. Determination of Surface Topography Images and Roughness Values (R_a_)

An Explorer Atomic Force Microscope (AFM) (Veeco, Cambridge, UK) was used to obtain topographic images and *R_a_* measurements (average deviation from a mean centre line), in non-contact mode, using a cantilever with a spring constant of 50 N m^−1^. Three images, from surfaces at a 5 µm × 5 µm scan size, from differently produced samples were taken to calculate the surface roughness measurements (*n* = 3).

### 2.3. Hardness of the Coatings

A Micro Materials Nanotest Nanoindentor (Micro Materials Limited, Wrexham, UK) was used to collect the nanoindentor results, using a Berkovich diamond. The diamond had a maximum to minimum load of 10–0.5 mN and maximum depth of 50 nm (*n* = 3).

### 2.4. Wettability of the Surfaces

A goniometer was used to measure the water contact angles of the surfaces. The sample was placed on a stage, and 5 µL of sterile distilled water was placed on the sample surface using a 5 µL micro syringe (Hamilton, Bonaduz, Switzerland) (*n* = 5). 

### 2.5. Preparation of Conidia

The *Aspergillus* species were inoculated from a short-term agar slope onto an agar plate using a sterile swab dipped into Sabouraud broth (Lab M, Bury, UK). The inoculated agar was incubated for 4 days at 29 °C. Five millilitres of Sabouraud broth was added to the fungal culture and using a sterile glass Pasteur pipette, the conidia were removed from the culture by rubbing the surface. A sterile beaker with a sterile magnetic stirrer was used to collect 5 mL aliquots of conidia, and the suspension was stirred for 30 min to separate the spores. The suspension was filtered through glass wool (VWR, Poole, Dorset) then conidia in the filtrate were harvested at 3000 g for 10 min. The conidia were washed three times in sterile distilled water and re-suspended to an optical density of 1.0 at 610 nm which equated to approximately 5.0 ± 0.3 × 10^6^ spores cm^2^ for *A. niger* 1957 and 5.3 ± 0.6 × 10^6^ spores cm^2^ for *A. niger* 1988. A haemocytometer was used to determine the number of conidia. The suspensions were stored at 4 °C and used within 4 weeks.

### 2.6. Imaging of Fungal Spores

Ten microliters of washed conidia were applied to a 1 µm × 1 µm polished silicon wafer (Montco Silicon Technologies, Spring City, PA, USA) and dried for 1 h in a Class 2 flow hood. The samples were transferred to 4% *v*/*v* glutaraldehyde (Agar Scientific Ltd., Stansted, UK) made with sterile distilled water which was stored at 4 °C for 24 h, before removal and washing in sterile distilled water. Following drying, the surfaces were removed and subjected to an ethanol (Fisher Scientific, Loughborough, UK) gradient in concentrations of 30% *v*/*v* in sterile distilled water for 10 min to 50% *v*/*v*, 70% *v*/*v*, 90% *v*/*v,* and finally 100% *v*/*v*. The samples were then stored in a desiccator until use. The samples were gold sputter coated. This was carried out using a Polaron E5100 sputter coater (Quorum, East Sussex, UK) at 0.09 mbar, for 3 min, at 2500 V, in argon gas at a power of 18–20 mA. Images of substrata were obtained using a JEOL JSM 5600L SEM (*n* = 3).

### 2.7. Perpendicular Force Measurements

Tipless cantilevers (Veeco, Cambridge, UK) were glued onto cantilever stubs (Veeco, Cambridge, UK) using a two-phase silver mounting adhesive. A glass cover slip (20 cm × 20 cm) was attached to an AFM mounting disc (Veeco, Cambridge, UK) using double-sided sticky tape. Ten microliters of washed spore suspension were placed onto the glass coverslip and dried in air for 1 h in a Class 2 flow hood. Cyanoacrylate gel (Bostik, Leicester, UK) was added to the coverslip. The attached coverslip on the mounting disc was placed onto the AFM stage, then the AFM camera and XY automated translation stage were used to move the tipless cantilever to the edge of the cyanoacrylate gel. The tip was lowered in the z plane until the cantilever momentarily touched the gel, and then the cantilever was immediately moved in the z plane away from the gel. The cantilever was moved across the coverslip until a suitable conidium was found. The cantilever was lowered until it touched the conidium. Once the gel had made contact with the conidium, the cantilever was left in place for 10 s before being lifted. The cantilever was removed from the AFM and left for 24 h to allow the adhesive to fully cure. Light and electron microscopy were used to check the quality and validity of the conidium glued to the cantilevers. Following quality control of the force data and cantilever spring constant, it was found that the conidium-cantilevers could each be used for up to twenty force measurements before they became unstable. The spring constant of the cantilever was determined before each perpendicular or lateral force experiment using the NanoScope AFM software v6.13. This was carried out by measuring the mechanical response of the cantilever to thermal noise as a function of time. To determine the perpendicular force of attachment between the particle probe and the substratum, the particle probe was brought into contact momentarily with the surface and the measurements calculated from force–distance curves where the distance travelled by the particle probe was plotted against the deflection of the cantilever. The deflection of the cantilever was converted into force (*F*) using Hooke’s law:

(1) F=−k×d

where *d* was the deflection of the cantilever and *k* the cantilever spring constant. By plotting *F* as a function of (*z*–*d*), the curve was corrected, where *z* was the vertical displacement of the piezoelectric scanner. The spring constant was multiplied using Hooke’s Law to calculate the force required to retract the particle probe from the surface. By subtracting the reciprocal of the slope, the displacement was calculated. The Hertz model was used to calculate Young’s modulus. The applied force was calculated by subtracting the zero of the force from the image setpoint and converted to nN from nA [23]. Ten replicates of each spore type were taken per surface sample.

### 2.8. Lateral Force Measurements Using Fungal Spores

One hundred microlitres of conidial suspension was pipetted onto the surface and dried for 1 h in a Class 2 hood. The AFM was used in contact mode and the cantilevers (pyramidal MLCT probes with a 35° angle) were calibrated before each use. One millilitre of sterile distilled water was added to the surface and the laser realigned. Lateral force assays were carried out at a speed of 1 Hz with a scan size of 50 μm × 50 μm. The number of spores remaining on the scanned surface was counted following each scan and the percentage calculated. Five separate scans and measurements were carried out per conidial type per sample. To determine the lateral force measurements, the applied force normal to the plane of interaction was calculated where Θ and *φ* were the probe geometry and cantilever orientation, respectively [32]:

(2) $$F_{app}=kd\times \sin\left(\Theta +\varphi \right).$$

The lateral force was determined using Equation (3) [32]:

(3) $$F_{lat}=F_{app}\times \cos(\Theta )$$

### 2.9. Attachment Assays

Replicate substrata (1 cm × 1 cm) were attached to a stainless steel tray using adhesive gum (Bostick, Leicester, UK) which was placed vertically in a Class 2 flow hood. A spore suspension at an OD of 1.0 ± 0.1 was sprayed onto the surface using an airbrush set to the finest spray setting (Badger Airbrush, London, UK), propelled by a Letraset 600 mL liquid gas canister (Esselte Letraset Ltd., Kent, UK). The distance between the spray and the airbrush was 10 cm and the speed of the spray was 50 mm s^−1^ with a flow rate of 0.2 mL s^−1^ as it was passed ten times from left to right. Following spraying, the substrata were held and rinsed once with 5 cm^3^ of distilled H_2_O, to remove loosely attached spores.

### 2.10. Adhesion Assays

For the adhesion assay, the replicate substrata were prepared as for the attachment assay but following spraying, the tray containing the substrata was laid horizontally and air-dried without rinsing.

### 2.11. Retention Assays 

Spores were prepared as previously and then 20 mL of spore suspension was pipetted onto three replicate substrata which had been placed horizontally in a glass Petri dish. These were incubated at room temperature for 1 h without agitation, then the test pieces were removed and rinsed once and dried as in the adhesion assay. All the substrata with spores were stained for 2 min using 0.03% acridine orange in 2% glacial acetic acid (Sigma, Dorset, UK), rinsed then air-dried and visualised and determined using epifluorescence microscopy (Nikon Eclipse E600, Nikon, Surrey, UK). 

### 2.12. Multifractal Analysis (MFA)

**Figure 1:**
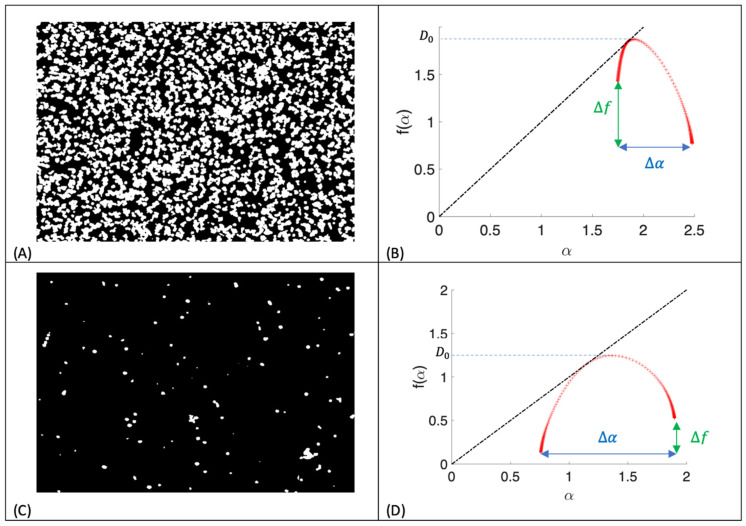
Binary images and multifractal spectra. (**A**) A binary image of *A. niger* 1957 on the cationic surface following the retention assay. (**B**) Multifractal $f\left(\alpha \right)$ curve for image (**A**). Density is measured by the fractal dimension, $D_{0}=1.87,$ a measure of dispersion is given by Δα=0.73, and a measure of clustering is given by Δf=0.66, indicating clustering of cells. (**C**) A binary image of *A. niger* 1957 on the cationic surface following the attachment assay. (**D**) Multifractal $f\left(\alpha \right)$ curve for image (**C**). Density is measured by the fractal dimension, $D_{0}=1.25,$ a measure of dispersion is given by Δα=1.14, and a measure of clustering is given by Δf=−0.39, indicating clustering of gaps. The black dash dot lines are $f\left(\alpha \right)=\alpha$.

The epifluorescent images of the spores were converted to black (surface) and white (cells) binary files using the MathWorks Image Processing Toolbox, where black pixels were assigned values of zero and white pixels one (Figure 1A,C). Multifractal analysis was carried out on both sets of images and the computed $f\left(\alpha \right)$ multifractal curves plotted. Images of each of the surfaces with retained microorganisms were processed and the averages were calculated. The numerical multifractal spectra were computed for −10≤q≤10, in all cases, and boxes of sizes 4, 8, 16, 32, 64, 128, and 256 were used in the computation of box-counting dimension, from which the $f\left(\alpha \right)$ curves were generated. This enabled the relative density, dispersion, and clustering of the white pixels to be computed. Two binary images and the corresponding multifractal $f\left(\alpha \right)$ curves are shown (Figure 1), where numerical measures of density $D_{0},$ dispersion Δα, and clustering Δf are also given. For a gentle introduction to fractals and multifractals, see [33]; for the mathematical theory behind multifractals, see [34]; and for a more detailed example in surface morphology, see [35].

### 2.13. Statistical Analysis

Statistical analysis was determined using a Student *t*-test, whereby significance was determined if *p* < 0.05.

## 3. Results

**Figure 2:**
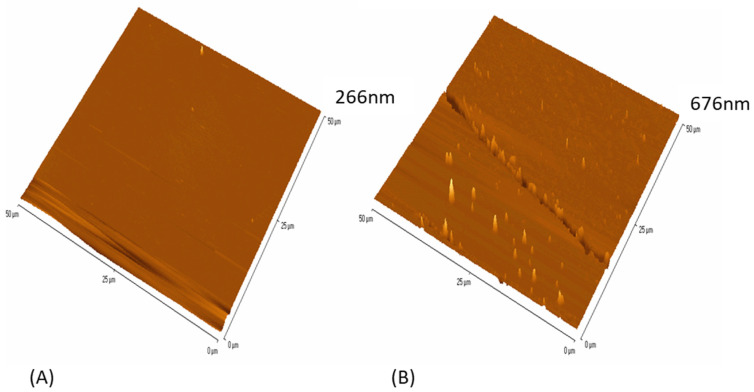
Surface topography of the (**A**) anionic and (**B**) cationic surfaces using a 50 µm × 50 µm scan.

AFM was used to demonstrate the topography of the anionic and cationic surfaces (Figure 2A,B). Apart from some linear features on the surfaces, the cationic surface also showed some small surface peaks. The z height in the images of the anionic surface was much lower (266 nm) (Figure 2A), whereas the z height of the cationic surface was 676 nm (Figure 2B) and this was representative across the surfaces (±20 nm).

To determine the effect of the surface properties on the binding and force of binding of the conidia using different methodological conditions, the hardness, *R_a_,* and wettability measurements of the anionic and cationic surfaces were assessed (Table 1). The results demonstrated that there were no significant differences in the results for the hardness values of the surfaces (*p* > 0.05) or for the *R_a_* values of the surfaces (*p* > 0.05). However, significant differences were demonstrated in the wettability on the surfaces (*p* < 0.05) (anionic 55°; cationic 37°).

**Figure 3:**
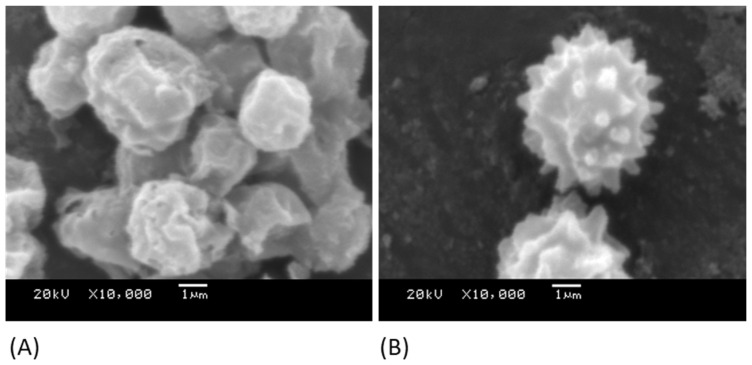
SEM images of the conidia of (**A**) *A. niger* 1957 and (**B**) *A. niger* 1988.

The conidia of *A. niger* 1957 (Figure 3A) and *A. niger* 1988 (Figure 3B) were imaged to demonstrate their differences in their surface features. It was demonstrated that the *A. niger* 1957 conidia, although wrinkled in appearance, did not have the spikey protrusions demonstrated by the conidia of the *A. niger* 1988. The mean sizes of the conidia spores were between 5.5 and 6 µm in diameter.

### 3.1. Perpendicular force measurements

**Figure 4:**
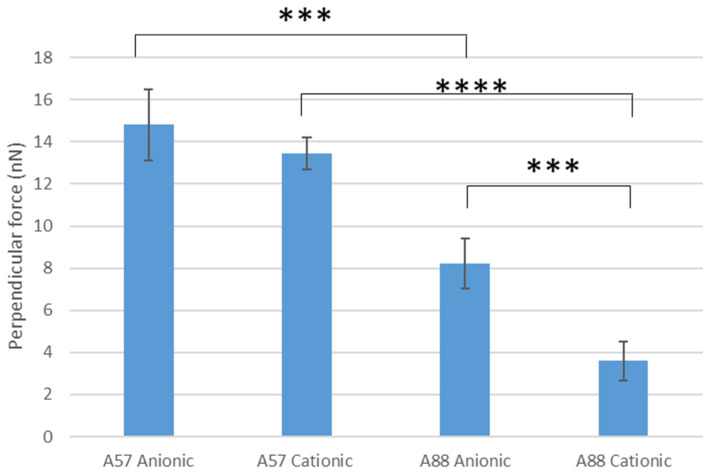
Perpendicular force measurements of the conidia on the anionic and cationic surfaces. A57 = *A. niger* 1957, A88 = *A. niger* 1988, *** = *p* < 0.005, **** = *p* < 0.001.

Perpendicular force measurements using cantilevers modified with conidia were used to determine the force of attachment of the spore to the surfaces (Figure 4). The highest perpendicular force was demonstrated by *A. niger* 1957 on anionic surfaces, whilst the least was shown by *A. niger* 1988 on the cationic surface. On the anionic surface, it was demonstrated that there was a significant difference in the amount of perpendicular force for the *A. niger* 1957 force of conidia attachment when compared to the *A. niger* 1988 on the anionic (*p* < 0.005) or cationic surface (*p* < 0.001). There was also a significant difference in the results for the *A. niger* 1988 when compared between the anionic (8.2 nN) and cationic (3.6 nN) surfaces (*p* < 0.005), however, there was no significant difference for the *A. niger* 1957 when compared between the anionic (14.8 nN) and cationic (13.4 nN) surfaces (*p* > 0.05).

### 3.2. Lateral Force Measurements

**Figure 5:**
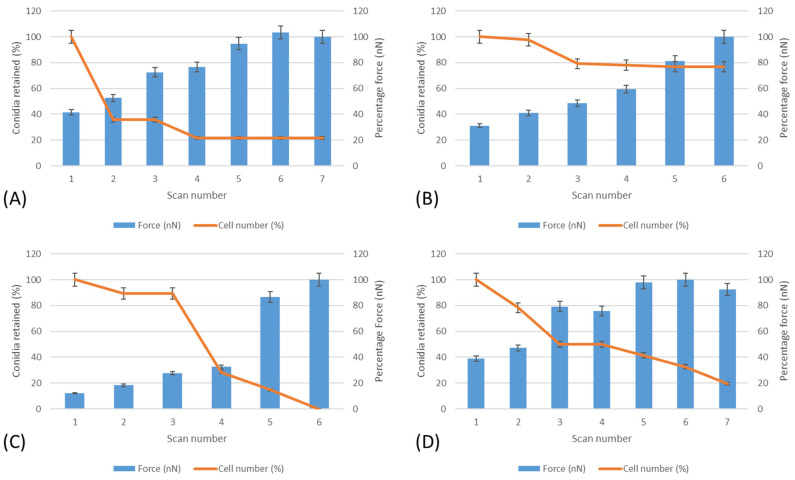
Lateral force measurements and the effect on conidial removal from the surfaces. (**A**) *A. niger* 1957 on anionic surfaces, (**B**) *A. niger* 1957 on cationic surfaces, (**C**) *A. niger* 1988 on anionic surfaces, and (**D**) *A. niger* 1988 on cationic surfaces.

Lateral force measurements were carried out on the surfaces to determine the force needed to push conidia from the surfaces (Figure 5). It was determined that *A. niger* 1988 on anionic surfaces were the most easily removed, whilst *A. niger* 1957 on the cationic surfaces were the most difficult spores to remove. 

### 3.3. Attachment, Adhesion, and Retention Assays

**Figure 6:**
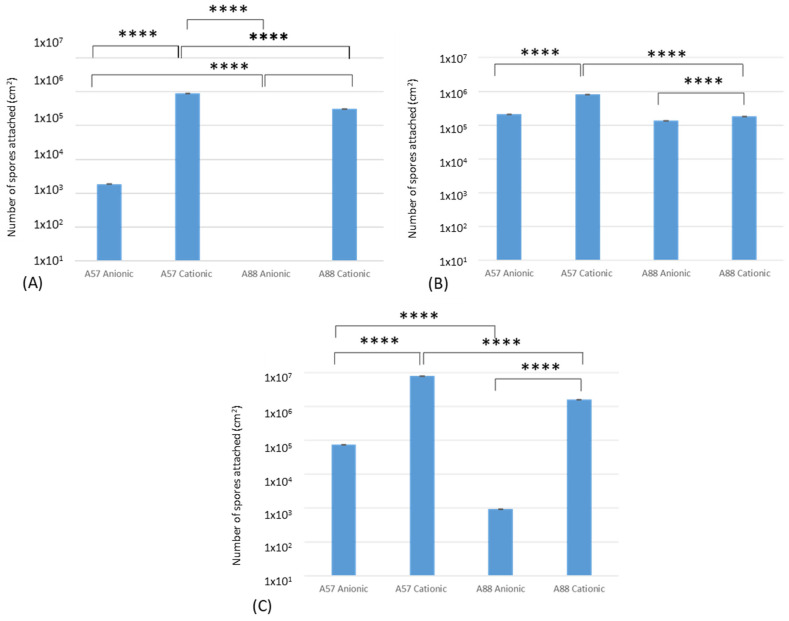
Conidia cm^2^ on anionic and cationic surfaces after (**A**) attachment, (**B**) adhesion, and (**C**) retention assays. A57 = *A. niger* 1957, A88 = *A. niger* 1988, **** denotes statistical differences whereby *p* < 0.001.

Following the attachment (Figure 6A), adhesion (Figure 6B), and retention (Figure 6C) assays, the results demonstrated that the greatest numbers of spores following the binding assays for *A. niger* 1957 on the cationic surfaces were 8.9 × 10^4^; 8.4 × 10^4^; 7.8 × 10^5^ cm^2^, respectively, and the least for *A. niger* 1988 on the anionic surfaces (0.0; 1.4 × 10^4^; 9.4 × 10^1^ spores cm^2^, respectively). There was a significant difference between the attachment, adhesion, and retention of *A. niger* 1957 on the anionic and cationic surfaces, *A. niger* 1957 and *A. niger* 1988 on the cationic surfaces, and *A. niger* 1988 on the anionic and cationic surfaces. In addition, for the attachment assays there was a significant difference in the numbers of *A. niger* 1988 on the anionic surface compared to the *A. niger* 1957 on the anionic and cationic surface and also when compared to *A. niger* 1988 on the cationic surfaces.

Following the adhesion assays, significant differences were determined. These were seen between *A. niger* 1957 and the anionic and cationic surface, *A. niger* 1988 and the anionic and cationic surface, and *A. niger* 1957 and *A. niger* 1988 and the cationic surface.

The retention assays demonstrated significant differences between *A. niger* 1957 and *A. niger* 1988 on the anionic surfaces (*p* < 0.001). The assays demonstrated the same pattern whereby *A. niger* 1957 on cationic surfaces > *A. niger* 1988 on cationic surfaces > *A. niger* 1957 on anionic surfaces and *A. niger* 1988 on anionic surfaces.

### 3.4. Multifractal Analysis (MFA)

**Figure 7:**
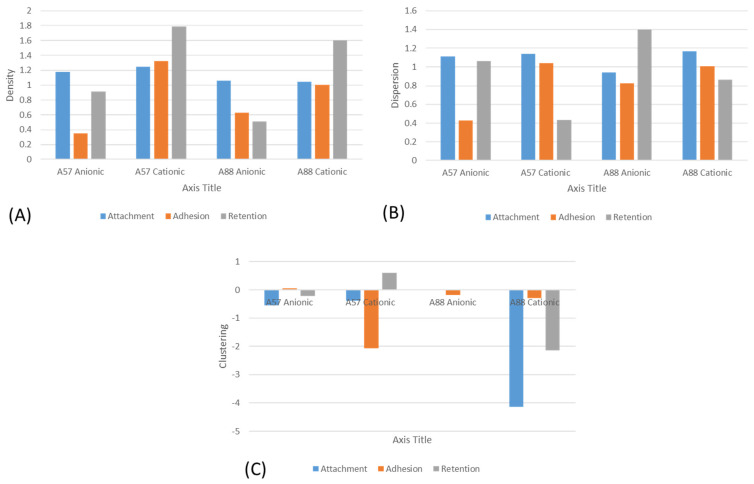
Multifractal analysis of the (**A**) density, (**B**) dispersion, and (**C**) clustering of the conidia on the surfaces.

The results from the multifractal analysis (Figure 7) demonstrated that both the *A. niger* 1957 and *A. niger* 1988 were most densely distributed across the cationic surfaces, regardless of the assay used (attachment, adhesion, or retention). Both the *A. niger* 1957 and *A. niger* 1988 were more evenly dispersed on the cationic surfaces following the attachment and adhesion assays, whereas they were more evenly dispersed on the anionic surfaces following the retention assays. The results for the clustering were the most difficult to elucidate in that all the *A. niger* 1988 conidia following the attachment, adhesion, or retention assays were the most clustered on the anionic surfaces and for the *A. niger* 1957 following the adhesion assays. However, following the attachment or retention assays, the conidia were more highly clustered on the cationic surfaces.

## 4. Discussion

One way to combat the transmission of organisms from fomites to patients has been the use of antiadhesive polymeric coatings. *A. niger* is an opportunistic pathogen and can form biofilms on polymeric structures [15,16,17]. The properties of a surface that affect the binding of conidia are still poorly understood, and the conidia of *Aspergillus niger* spp. are still of concern in terms of the potential development of infections on biomaterials and from surface transmission. 

In this work, there were no significant differences in the results for the hardness or the *R_a_* values of the surfaces. However, significant differences were demonstrated in the wettability of the surfaces. In addition, although of similar sizes, SEM imaging of the conidia of the *A. niger* 1957 and *A. niger* 1988 demonstrated them to be of different morphologies, whereby the *A. niger* 1957 had a round shape with wrinkled surfaces, whilst *A. niger* 1988 demonstrated spikey protrusions from the surface, thus adding a further complexity to this work.

### 4.1. Perpendicular and Lateral Force Measurements

Perpendicular force measurements may be used to determine the force of attachment of conidia to a surface. The highest perpendicular force measurement was demonstrated by *A. niger* 1957 and *A. niger* 1988 on anionic surfaces which was the less wettable surface. This finding is in agreement with work carried out on the strength of attachment of the spores on several surfaces which found that perpendicular force measurements could be related to the wettability of the surfaces [23]. Lateral force measurements were carried out on the surfaces to determine the force needed to push conidia from the surfaces. Both the *A. niger* 1957 and *A. niger* 1988 were more difficult to remove using the lateral force measurements from the anionic surface. This is in agreement with work carried out on PMMA surfaces, whereby *A. niger* 1988 spores were the most difficult to remove from a p(γ-MPS-co-LMA) spin-coated surface which was also the least wettable surface [25]. This similarity in results for the perpendicular and lateral force measurements may have occurred since in both instances a force was applied, and the surfaces and conidia were similar in their surface properties. Although the negatively charged conidium will be attracted to the positively charged cationic surface, they could be more easily removed due to the forces enabling effects from the Stern layer to come into play, thus resulting in the ease of removal of the conidium from the cationic surface.

### 4.2. Binding Assays and Multifractal Analysis

The results of the binding assays (attachment, adhesion, or retention) demonstrated that both the *A. niger* 1957 and *A. niger* 1988 conidia bound in greater numbers to the cationic surfaces and they were also found to be distributed in the highest density across these surfaces. Given that the conidia were of different shapes, this result negates in this instance that the shape of the conidia influenced the results. It may be speculated that the attachment of the conidia to the surfaces was influenced by electrostatic forces. Fungal spores generate an electrical surface charge, and it has been suggested that this may have a function in spore–surface attraction [36]. It is also known that *Aspergillus* spp. have on their surface small amphipathic proteins which are known as hydrophobins and these can self-assemble at water–air interfaces [17,37]. It is thought that hydrophobins may be responsible for conidia binding to surfaces [38,39,40]. It is also known that the outside of the conidia of *A. niger* is composed in part of proteins and lipids [21,41], although a small amount of carbohydrate is also present. *A. niger* also contains melanin pigments which influence the net surface charge of the conidia due to the incomplete dissociation of carboxyl groups, and these pigments are found on top of the outer spore wall layer [42]. In agreement with these findings, diffuse reflectance for infrared Fourier transform spectroscopy (DRIFTS) demonstrated that both types of *A. niger* spores demonstrated broad hydrogen-bonded -OH stretching bands, C-H stretching vibrations ester carbonyl bands, amide I and II carbonyl bands, and C-O-C bending vibrations [43]. Work by Grunér [44] demonstrated that hydrophobins bound to cationic but not anionic surfaces immersed in aqueous solution and that it was electrostatic forces that were important for the interaction between the hydrophobins and the polar surface. They speculated that on the hydrophobic surface, the hydrophilic side of the layer was turned toward the solution, and on the cationic surface the more hydrophobic side of the layer was turned toward the solution [44,45].

In agreement with the findings of our binding assays, work by others has shown similarities with a variety of cells and biomolecules. It has been demonstrated that protein adsorption and macrophage adhesion on cationic surfaces was higher than on zwitterionic and anionic polyurethanes [46]. The effect of biofouling to charged polymers has been investigated by using cationic and anionic brushes to test their resistance to protein adsorption, bacterial and green alga zoospore adhesion, and settlement of barnacle larvae, and it was found that anionic surfaces showed lower biofouling due to a higher surface wettability and repulsive electrostatic interactions between the anionic polymer and the negatively charged bacterial cell wall [47]. However, in contrast, Yang et al. [48] demonstrated that anionic poly(sodium styrene sulfonate) did not reduce bacterial attachment as effectively as zwitterionic or neutral polymers [48].

The tendency to form conidial aggregates at the early stage of cultivation by *A. niger* has long been recognized [42] and the aggregation behaviour has been attributed to electrostatic surface properties [44,49,50,51,52]. The use of mathematical analysis can be used to provide further information on the density, dispersion, and clustering of microorganisms across a surface. The theory and applications of MFA have been described [53,54]. The results from the multifractal analysis demonstrated that both the *A. niger* 1957 and *A. niger* 1988 were most densely distributed across the cationic surfaces, regardless of the assay used (attachment, adhesion, or retention). The dispersion of conidia across the surfaces was found to be more homogeneous on the cationic surfaces following the attachment and adhesion assays whereby the conidia were forced onto the surface via the spraying assays, whereas following the retention assays the conidia were more homogeneously dispersed across the anionic surfaces. One of the key surface properties that is thought to contribute to microbial binding to a surface is substratum hydrophobicity [52], and this may have affected the dispersion of the spores across the surfaces.

The results from the clustering assays were more difficult to explain. However, a clear trend was observed with the *A. niger* 1988 conidia whereby the greatest spore clustering was observed on the anionic surfaces and the cationic surfaces following the use of the *A. niger* 1957 conidia after the attachment and retention assays, suggesting that the spore shape influenced the pattern on clustering. The effect of spore shape has also been demonstrated by Whitehead et al. [55], whereby in the presence of a washing step, both the properties of the surfaces and the conidia affected conidial adhesion and retention. 

The disparity in the results between studies may be explained in part due to the ranges of the properties of the surfaces tested. For example, when PVAc and PVOH moulded surfaces were used to determine the binding of *A. niger* 1957 and 1988 conidia to surfaces and following adhesion and retention assays it was found that in contrast to the results presented here, the more polar surface retained all the types of conidia and that binding to the surfaces was influenced by the wettability of the surfaces and spores. However, the more polar surfaces were significantly rougher in the Liauw study [56]. Such position-dependent interactions give a theoretical equilibrium distance at which the binding between the spores is most stable [44]. Hence, different systems, depending on their individual properties, will act in a variety of ways since the equilibrium distance between two interacting bodies will be a question of the entirety of reversible interactions. These interactions are complex and will involve a number of facets [44], the most important of which will become apparent depending on the variation in the surface properties.

## 5. Conclusions

The highest perpendicular force (i.e., strongest attachment) was demonstrated by *A. niger* 1957 and *A. niger* 1988 on anionic surfaces. Both conidial types were more difficult to remove using the lateral force measurements from the anionic surface, although fewer spores remained on the anionic surfaces after the physical treatments of the traditional washing assays. Dispersion of conidia across the surfaces was more homogeneous on the cationic surfaces following the attachment and adhesion assays, whereas following the retention assays the conidia were more homogeneously dispersed across the anionic surfaces. The results of the binding assays (attachment, adhesion, or retention) demonstrated that both the *A. niger* 1957 and *A. niger* 1988 conidia bound in higher numbers to the cationic surfaces and they were also found to be distributed most densely across these surfaces. The use of such a wide range of analytical tools and methods illustrates the complexity of the interactions between the spore and the surface. The results suggest that in this instance, surface wettability was a major factor in influencing conidial interactions with the surface. Further, this work demonstrated that there were more tightly bound conidia that remained after physical and washing treatments and these are important since these retained spores are likely to be those which can proliferate to create biofilms and colonies. This observation indicates the next direction for study, namely removing these tightly bound conidia and observing structural or surface differences between these and other conidial genus. 

## Figures and Tables

**Table 1:** Hardness, *R_a_,* and contact angle measurements of the anionic and cationic surfaces.

	Nanoindentation (GPa)	*R_a_* (nm)	Contact Angle (°)
Anionic	0.7 ± 0.2	2.4 ± 0.6	55 ± 0.8
Cationic	0.5 ± 0.01	21.9 ± 11.5	37 ± 3.1

## Data Availability

Original/source data for the figures and data in the paper are available from the lead contact. Processed data are supplied and can be accessed from MMU e-space (https://espace.mmu.ac.uk/629087/, accessed on 25 September 2022).
